# Impact of PA-100 AST System Rapid Antibiotic Susceptibility Test on Antibiotic Prescription for Community-Acquired Urinary Tract Infections in Spanish Primary Care Settings

**DOI:** 10.3390/antibiotics15050520

**Published:** 2026-05-21

**Authors:** Lourdes Martínez-Berganza Asensio, Gonzalo Largo-Rojo, Ana Isabel Menéndez-Fernández, Carmen Solano-Villarrubia, María Fuentes-Romero, José Medina-Polo

**Affiliations:** 1 Centro de Salud Ensanche de Vallecas, Servicio Madrileño de Salud, 28051 Madrid, Spain; 2 Department of Urology, Hospital Universitario 12 de Octubre (imas12), 28041 Madrid, Spain

**Keywords:** cystitis, urinary tract infection, antimicrobial resistance, primary care, antibiotic treatment

## Abstract

**Background/Objectives**: This intervention study compared the impact of the PA-100 AST System (PA-100) with the standard of care on antibiotic-prescribing behaviour for community-acquired urinary tract infections in a Spanish primary care setting. **Methods**: Women seeking care for symptoms of uncomplicated urinary tract infections were recruited based on the last digit of their regional personal identification number in a control (no PA-100 result available) or intervention (PA-100 result available) arm. Differences in antibiotic-prescribing behaviour were analysed using Fisher’s exact test, with the sample size powered to detect a change in prescription in ≥6% of patients. **Results**: Availability of the PA-100 revealed resistance to fosfomycin in 21.5% of confirmed infections. This significantly shifted prescription away from fosfomycin towards nitrofurantoin and amoxycillin/clavulanic acid (*p* < 0.001). In accordance with local guidelines, fosfomycin was the most frequently prescribed antibiotic in the control arm (65.9%), whereas a significantly lower rate (37.7%) was observed in the intervention arm. **Conclusions**: The PA-100 shows potential to support antimicrobial stewardship by enabling targeted antibiotic treatment at the first visit and improving care in primary care settings.

## 1. Introduction

Uncomplicated urinary tract infections (uUTI), i.e., localised cystitis, according to the most recent recommendations by the EAU [1], are common community-acquired bacterial infections, regularly treated at the primary care (PC) level [2], where they account for 18.4% of all visits related to infection episodes, second only to respiratory infections [3]. This makes UTI one of the most common diseases requiring antibiotic treatment.

Current diagnosis and treatment of uUTI, according to guidelines [4], is fundamentally based on symptoms. Urine analysis using dipsticks or culture is not mandatory unless patients present with atypical symptoms or upon failure of previous antimicrobial therapy [4]. However, urine dipstick tests are widely used as a diagnostic decision support tool. Current guidelines describe the most common bacterial species causing uUTI and the antibiotics to which they are usually susceptible. Therefore, treatment of uUTI in PC is generally done empirically, which has been reported to lead to inappropriate antibiotic treatment in up to 50% of cases [5]. This includes excessive prescription of antibiotics for asymptomatic bacteriuria cases [6].

Efforts have been made to implement antibiotic stewardship programmes that involve all stakeholders, including institutions, clinicians, nurses, and patients. The recommendations of the Spanish antimicrobial stewardship programme (PROA) propose the prescription of fosfomycin (single dose) to treat patients with urinary symptoms or nonspecific symptoms and a positive dipstick test [7]. Such programmes create pressure to reduce inappropriate (unnecessary or ineffective) antibiotic prescriptions for UTI.

Adherence to the guideline recommendations can vary and is often suboptimal in certain PC settings, thereby exacerbating the negative effects of antibiotic mismanagement on antimicrobial resistance (AMR) [8]. Mismanagement of uUTI can lead to increased treatment failure rates, higher costs to the healthcare system [9] and recurrent UTI episodes which are frustrating for patients and may negatively affect their quality of life [10]. While overtreatment of suspected UTI episodes fuels AMR, undertreatment can lead to serious worsening of a patient’s health, highlighting the need for better diagnostic tools [11].

It has been widely acknowledged that proper management of AMR requires intensive use of appropriate diagnostic tests, emphasising the role of diagnostic stewardship [12]. Rapid diagnostics available at the point of care (POC) are needed to correctly manage UTI. In the absence of alternatives, urine dipsticks are commonly used as POC tests, despite limited accuracy in detecting UTI [13]. Hence, new technologies that optimise management of UTI are needed, as shown in the early value assessment by the National Institute for health and Care Excellence (NICE) in the UK of several tests, including the PA-100 AST System [14].

The Sysmex PA-100 AST System (PA-100) has CE-IVD certification and identifies bacteriuria in fresh urine samples within 15 min. In positive samples, it performs phenotypic antimicrobial susceptibility testing (AST) in an additional 15–30 min. The potential of this system to reduce the inappropriate use of antibiotics against resistant bacterial strains was demonstrated in earlier investigations that focused on the diagnostic accuracy of the analyser [15,16], which led to Sysmex Astrego winning the prestigious Longitude Prize on AMR in 2024 for the PA-100. However, this benefit remains largely hypothetical, and the actual impact of the system on treatment decisions has not yet been described. The aim of this study was to implement the PA-100 in a real-world clinical setting and to evaluate whether clinicians treating patients with UTI would alter their treatment decisions and choice of antibiotics when the PA-100 results were made available, compared with the current standard of care.

## 2. Results

**Figure 1:**
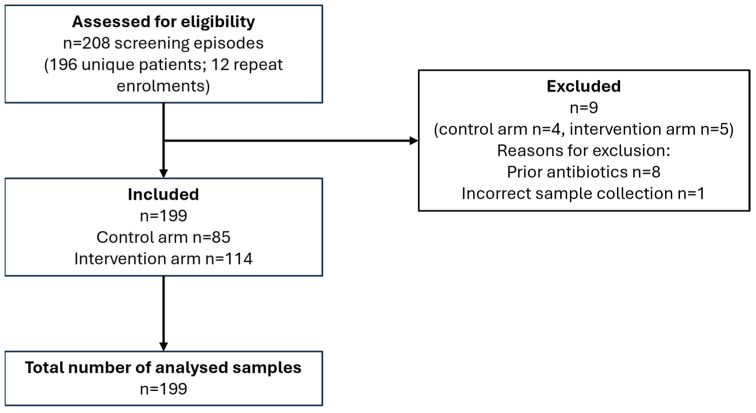
Schematic representation of the patient inclusion results.

A total of 196 patients were enrolled, 12 of whom presented to the PC twice during the study period. The reason for these repeat visits was not documented. These repeat presentations were enrolled as new samples, resulting in a total of 208 sample enrolments. Nine samples were excluded retrospectively due to either antibiotic treatment within the preceding 7 days or an analysis on the PA-100 being performed more than 30 min after sample collection. The final study sample therefore consisted of 199 samples, including 85 in the control arm and 114 in the intervention arm. Repeat visits were treated as independent events during analysis. The unequal sample sizes between study arms resulted from of the quasi-randomisation strategy, in which assignment was determined by whether the last digit of the personal identification number was even or odd. Figure 1 summarises patient recruitment and inclusion in the analysis, according to the criteria described above.

Patient characteristics and an overview of urine dipstick and PA-100 bacteriuria results are presented in Table 1. No overall significant difference in urine dipstick results was observed between the control and intervention arms (*p*-value 0.3241). In the intervention arm, 25 samples (21.9%) showed nitrate-positive dipstick results, of which 84% were PA-100 bacteriuria-positive. Samples that were PA-100 bacteriuria-negative showed significantly fewer positive nitrite dipstick results (*p*-value < 0.001). The analytical performance of the urine dipstick for the detection of bacteriuria is reported in the literature [13].

**Figure 2:**
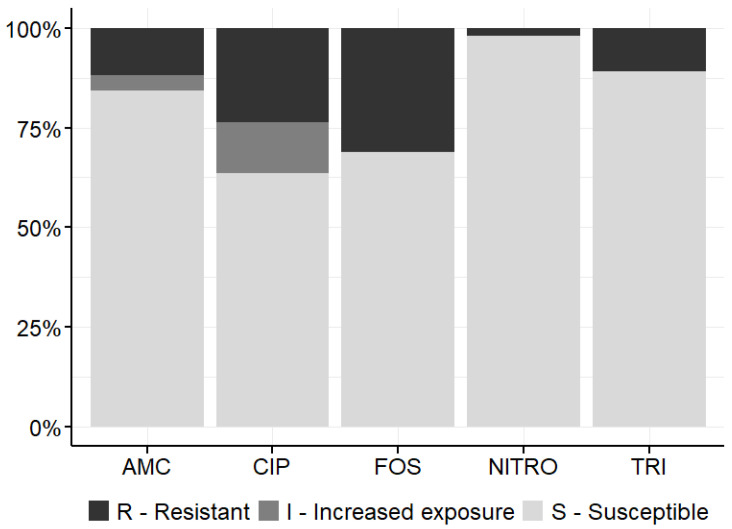
Relative frequency of PA-100 antibiotic susceptibility test results for amoxicillin/clavulanic acid (AMC), ciprofloxacin (CIP), fosfomycin (FOS), nitrofurantoin (NITRO), and trimethoprim (TRI) in the intervention arm. Low growth (LG), N/A and technical errors were excluded.

The PA-100 AST results of the 65 bacteriuria-positive samples are presented in Table 2 and the relative frequency is shown in Figure 2. Most bacterial strains were sensitive to the antibiotics tested in PA-100; high resistance rates were detected for fosfomycin (21.5%) and ciprofloxacin (20.0%).

**Figure 3:**
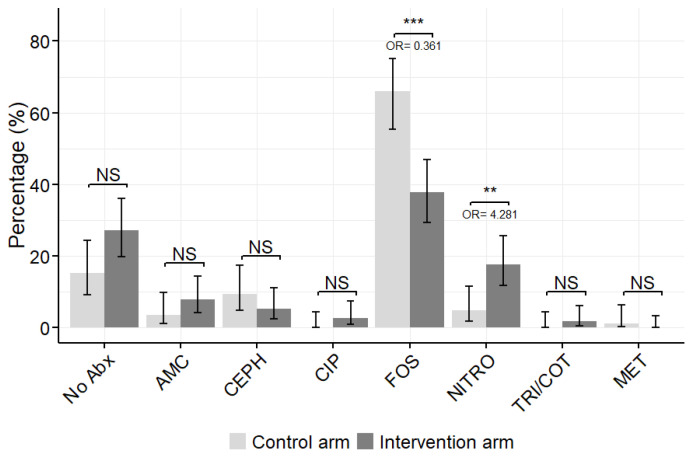
Relative frequency of antibiotic prescription in the control and intervention arms. Amoxicillin/clavulanic acid (AMC), cefixime and cefuroxime are combined as cephalosporine (CEPH), fosfomycin (FOS), nitrofurantoin (NITRO), ciprofloxacin (CIP), trimethoprim/cotrimoxazole (TRI/COT) and metronidazole (MET). The error bars represent 95% confidence interval. NS: Not significant, ** *p*-value < 0.01, *** *p*-value < 0.001.

The antibiotic prescription in the control and intervention arm is shown in Table 3 and Figure 3. The overall antibiotic prescription was significantly different between the control and intervention arm (*p*-value < 0.001). In accordance with local guidelines, fosfomycin was the most frequent antibiotic prescribed in the control arm (65.9%) and featured a significantly lower prescription rate of 37.7% in the intervention arm (*p*-value < 0.001). The nitrofurantoin prescription rate significantly increased in the intervention arm (from 4.7% to 17.5%, *p*-value 0.0073) compared to the control arm. No antibiotic prescription, or prescription of any of the other three antibiotics, showed no significant difference between the control and intervention arm.

Table 4 shows the PA-100 AST results for fosfomycin and the fosfomycin prescription rate. In the intervention arm, 43 patients were treated with fosfomycin, and 23 of these samples were susceptible to fosfomycin (53.5%), two samples resulted in an AST error for fosfomycin (4.6%) and 16 samples were PA-100 bacteriuria-negative (37.2%). In the latter two scenarios, clinical judgement prevailed and fosfomycin was described empirically according to the guidelines. Forty patients in the intervention arm were treated with one of the other six available antibiotics (35.1%). Of these 40 samples, 14 showed fosfomycin resistance (35.0%), five low growth (12.5%), eight samples were susceptible to fosfomycin (20.0%), and one sample was bacteriuria-negative (2.5%). Patients with fosfomycin-resistant strains were primarily treated with nitrofurantoin (64.3%), amoxicillin/clavulanic acid (21.4%) or ciprofloxacin (14.3%). One out of five patients with AST low-growth results for fosfomycin had a fosfomycin intolerance and was treated with nitrofurantoin.

## 3. Discussion

Increasing AMR has been identified as a major health threat by the global community. The WHO estimates that in 2050, failure of antibiotic intervention will become a major global health threat worldwide [17]. One of the drivers of AMR is overuse and/or misuse of antibiotics, which is frequently seen in empirical treatment of patients with uUTI in a PC setting. Since antibiotic susceptibility testing remains a specialised investigation for microbiology laboratories, the treating PC physician usually has no other choice than to prescribe an antibiotic without knowledge of the causative pathogen or the efficacy of the antibiotic against that organism. Local guidelines aim to provide actionable selection criteria based on reported resistance patterns in the area to avoid the further spread of resistance, but these measures are not that effective [5]. Clinical scores such as the Acute Cystitis Symptom Score [18] can also be used to guide antibiotic use. Additionally, diagnostic companies postulate benefits through the deployment of POC tests; however, while analytical performance is available for IVD cleared devices, their impact on patients, healthcare funders, and society is often unclear.

Current clinical guidelines, including the 2026 European Association of Urology guidelines [19], recommend that uncomplicated urinary tract infections in otherwise healthy women are primarily diagnosed and managed on the basis of clinical symptoms and urinalysis, with microbiological investigation and antimicrobial susceptibility testing generally reserved for recurrent, treatment-refractory, systemic, or otherwise complicated infections. This reflects current practice, where AST requires sample transport to a microbiology laboratory and leads to delayed results. The PA-100 now enables identification of growing bacteria and AST at the point of care, allowing targeted antibiotic treatment on the day of consultation. Targeted implementation in selected patient groups could enhance clinical outcomes and antimicrobial stewardship.

This is the first study to evaluate the impact of the PA-100 on clinical decision-making and antibiotic prescription in a PC setting among women presenting with symptoms of UTI. It highlights the well-understood limitations of empirical treatment but also demonstrates the potential that a POC AST device holds for the improvement in healthcare. However, to date, no studies have been able to link point-of-care AST with reduced rates of antimicrobial resistance. It remains unclear whether this is due to the lack of positive results or the absence of cost-efficient diagnostic options that would enable such a long-term investigation.

The overall antibiotic prescription rate was reduced by more than 10% in the intervention arm; however, this effect narrowly missed statistical significance (*p*-value 0.057). Several samples with LEU-positive dipstick results were PA-100-negative. However, it is unlikely that all of these samples were false negatives since leukocyte-positive and nitrite- negative dipstick results are often not correlated with a positive microbiology test [20,21], and the treating physicians were informed about the diagnostic limitations of the PA-100 before the start of the study, resulting in the treatment of those patients with antibiotics.

The PA-100 revealed a higher-than-expected resistance against fosfomycin in 21.5% of bacteriuria-positive samples. A POC device is not a microbiological reference; however, in a previous investigation, the PA-100 demonstrated 95.2% overall categorical agreement with a microbiological reference and specificity above 90% for all antibiotics in the test [15]. A comparison with other samples not included in this study but sent to a microbiology reference laboratory showed that the resistance pattern was most likely driven by higher resistance rates to fosfomycin compared to nitrofurantoin in *Escherichia coli* and *Klebsiella pneumonaie,* the predominant species in uUTI [22]. As previously reported in the literature, increasing resistance to fosfomycin in community-acquired infections has been observed, sometimes linked to the increase in ESBL prevalence [23,24]. While this does not provide certainty about the AST results in the study, it indicates a trend in local resistance patterns in the area that could support the findings in this study.

The attending clinicians took the PA-100 results into consideration for their treatment decision resulting in a significant change in the antibiotic-prescribing behaviour. The high frequency of fosfomycin resistance detected by the PA-100 was the single key element driving this change in prescribing behaviour, highlighting the drawback of empirical treatment. The attending clinicians reported that the availability of the PA-100 AST not only influenced the prescription of nitrofurantoin, but also amoxicillin/clavulanic acid, an antibiotic they would not normally prescribe. However, in the intervention arm, they considered its use when the PA-100 AST indicated susceptibility. Due to the sample size, however, this effect did not reach statistical significance. The predominant use of fosfomycin (78%) in a setting where approximately one-fifth of UTIs are resistant to this antibiotic may contribute to increasing resistance rates within the community. If the level of bacteriuria is below the PA-100 positivity threshold (>50,000 CFU/mL), bacterial presence may not be detected in samples from which a conventional microbiology laboratory might successfully isolate a strain. This is an inherent limitation associated with the short turn-around time of the PA-100 and was communicated to the participating clinicians beforehand. Consequently, the PA-100 did not significantly reduce the frequency of antibiotic prescription in the intervention arm, as clinicians continued to rely on clinical symptoms as a key component of treatment decision-making. In contrast, adherence to the AST result was complete, as the PA-100 has demonstrated high concordance with the microbiological reference in previous studies [15], allowing clinicians to trust its recommendation.

In accordance with current guidelines [19], the authors believe that the principal strength of the PA-100 lies in its AST functionality rather than in the general detection of bacteriuria. The long-term public health impact of such devices, particularly in reducing antimicrobial resistance within the community, remains to be established. This is one reason why AST is currently reserved for systemic or complicated infections, thereby helping to avoid the financial burden associated with diagnostic overutilization.

Antibiotic-prescribing behaviour may be influenced by behavioural factors, as described in the literature [25]. In the present study, any such influence could only have affected the binary decision of whether to prescribe antibiotics or not; however, no significant differences were observed in this regard. Instead, changes in antibiotic prescription were driven by the availability of AST results to the treating clinician.

A limitation of the study is that patients were not followed up to determine whether the prescribed antibiotics effectively cleared the infection. This could be addressed in a future study to evaluate the clinical impact of the PA-100. Without such microbiological follow-up, the appropriateness of the prescription changes in this cohort remains unvalidated against the reference method. Furthermore, the enrolled population may have included patients for whom antibiotic treatment was considered clinically indicated by the clinician, or those with a positive urine dipstick test. A diagnostic workflow integrating the PA-100 into a PC setting, with the aim of maximising patient benefit while ensuring cost effectiveness within the healthcare system, will need to be developed.

## 4. Materials and Methods

### 4.1. Study Design and Patient Population

This prospective, interventional study was conducted at Centro de Salud Ensanche de Vallecas (CSEV) in Madrid, Spain, a PC centre. Patients were recruited according to the following criteria: non-pregnant female, >18 years old, presenting with acute UTI symptoms that had been present for <7 days (symptoms according to the current guidelines [4]), with no antibiotic treatment within the preceding 7 days, positive urine dipstick for leukocyte esterase (LEU) and/or nitrites (NIT) and who provided a clean-catch mid-stream urine sample. Only patients with suspicion of localised cystitis between July 2024 and June 2025 were included. Exclusion criteria included the following: antibiotic treatment within 7 days prior to consultation with the doctor, urine sample not collected using the midstream clean-catch method, or any suspicion of complicated UTI (e.g., anatomical abnormality of the urinary tract, kidney disease or indwelling catheters).

Informed consent was obtained from all patients, as lack of consent was an exclusion criterion. The study was conducted according to the Declaration of Helsinki and approved on 16 May 2024 by the Ethics Committee of the Hospital 12 de Octubre, Madrid.

The study was retrospectively registered number NCT07189429 (date 16 September 2025).

### 4.2. Sample Size Calculation

The study was designed to detect an average change in antibiotic prescription frequency of 6% at a significance level of 0.05. The calculated sample size, based on similar bacterial resistance patterns as described previously [15], was 87 patients per group (control and intervention arms).

### 4.3. Urine Collection

A clean-catch mid-stream urine sample was collected from patients. In the intervention group, one aliquot of the sample was separated and used for the PA-100 assay.

### 4.4. Test Methods

#### PA-100

The PA-100 is an automated, cartridge-based urine analyser that detects bacteriuria in fresh urine samples (<30 min after voiding, sample volume 400 µL) within 15 min, and in positive samples (>50,000 CFU/mL) performs AST within a total of 45 min. The system tests resistance against 5 antibiotics simultaneously: amoxicillin/clavulanic acid, ciprofloxacin, fosfomycin, nitrofurantoin and trimethoprim. AST results are produced according to EUCAST and assigned one of the following susceptibility categories: S (susceptible), I (susceptible, increased exposure), R (resistant) or NA (not applicable).

A more detailed description of the technology is available elsewhere [15,26,27,28,29]. In previous studies the PA-100 demonstrated sensitivity and specificity values for the detection of bacteriuria of 84.0 and 99.4% respectively. The overall categorical agreement with the reference of the AST for the antibiotics tested, AMC, CIP, FOS, NITRO and TRI, was 89.6, 85.4, 94.0, 95.0 and 96.4%, respectively [15].

### 4.5. Study Design and Workflow

#### 4.5.1. Control Arm

Patients with a regional personal identification number ending in an even digit were enrolled in the control arm. Urine samples were analysed using dipsticks, and patients were treated according to current guidelines [4]. No PA-100 measurement was performed in the control arm.

#### 4.5.2. Intervention Arm

Patients with a regional personal identification number ending in an odd digit were enrolled in the intervention arm, and their urine samples analysed by dipstick and the PA-100. The treatment decision was made after results from the PA-100 were available; however, clinical criteria based on symptoms such as dysuria, urgency, frequency or suprapubic pain prevailed.

### 4.6. Study Workflow

**Figure 4:**
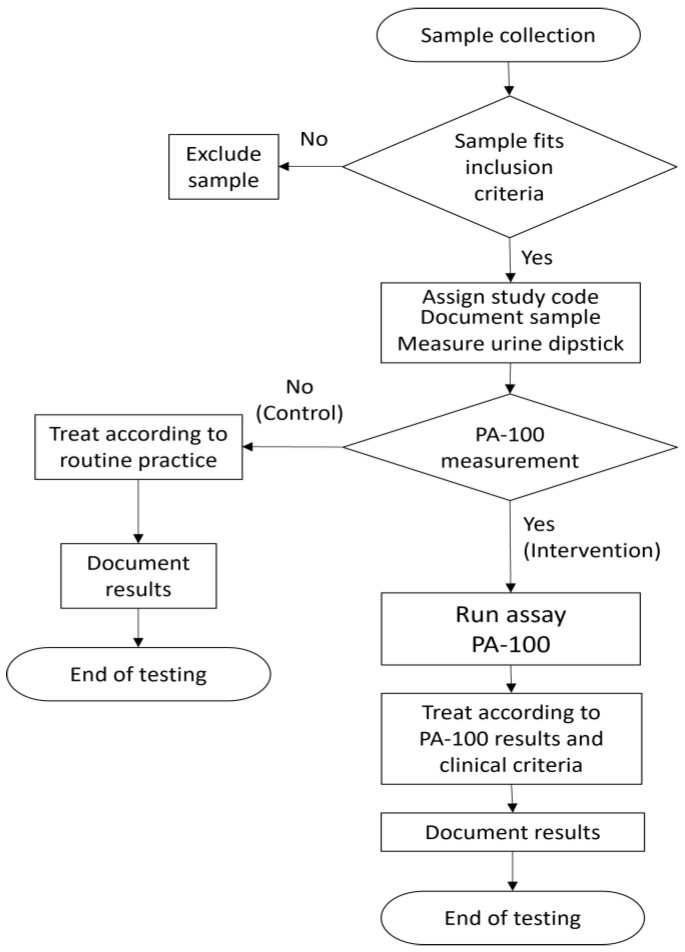
Schematic representation of the study workflow.

Figure 4 summarises the study workflow for the control and intervention arms. Nurses were responsible for patient interviews, documentation and measurements (dipstick and PA-100). Doctors consulted the patients and decided on prescription.

### 4.7. Statistical Analysis

Statistical analyses were performed using R Software version 4.4.1 (R Foundation for Statistical Computing, Vienna, Austria). Comparison of proportions was done with Fisher’s exact test and 95% confidence intervals (CIs) were calculated according to Wilson.

## 5. Conclusions

The PA-100 was successfully implemented in a PC setting, where it identified prevailing resistance patterns and provided clinicians treating patients with UTI with evidence-based, actionable guidance. Its use significantly reduced the prescription of ineffective antibiotics and shifted prescribing patterns away from fosfomycin towards nitrofurantoin and amoxicillin/clavulanic acid. However, the clinical impact of these prescription changes remains to be confirmed, as patients were not followed microbiologically to assess the infection clearance or to validate treatment appropriateness against the reference method. In addition, the study population may have included patients selected on the basis of clinical assessment or positive urine dipstick findings rather than microbiologically confirmed infection. While the PA-100 primarily supports evidence-based antibiotic selection, an equally important long-term objective of point-of-care diagnostics in primary care is to reduce unnecessary antibiotic prescription whenever infection is not microbiologically supported, or antibiotic treatment is not clinically indicated. The potential of the system to reduce overall antibiotic prescription, as well as its long-term impact on antibiotic resistance patterns, remains to be demonstrated. Further studies are therefore needed to evaluate clinical outcomes, optimise diagnostic workflows integrating the PA-100 into primary care, and determine its long-term impact on antibiotic-prescribing and antimicrobial resistance patterns.

## Figures and Tables

**Table 1:** Patient characteristics and urine dipstick results for leucocyte esterase (LEU) and nitrites (NIT) in the control and intervention arms. PA-100 bacteriuria results are shown for the intervention arm. The relative frequency of each parameter is given in parentheses. Statistical significance was calculated by comparing the control and intervention arms. Urine dipstick results for the intervention arm are further stratified according to the PA-100 bacteriuria result.

	Control Arm (n = 85)	Intervention Arm (n = 114)		*p*-Value
Age (median & 95% CI)	46.0 (42.0–52.0)	50.0 (44.0–54.5)		0.358
Previous use of painkillers	18 (21.2%)	24 (21.1%)		1.000
Diabetes mellitus	4 (4.7%)	9 (7.9%)		0.404
Urgency	43 (50.6%)	61 (53.5%)		0.774
Suprapubic pain	55 (64.7%)	70 (61.4%)		0.550
Fever	6 (7.1%)	12 (10.5%)		0.463
Flank pain	13 (15.3%)	17 (14.9%)		1.000
General discomfort	26 (30.6%)	51 (44.7%)		0.056
Nausea/vomiting	6 (7.1%)	12 (10.5%)		0.462
History of recurrent UTI	17 (20.0%)	16 (14.0%)		0.253
Duration of symptoms in hours (median & 95% CI)	48.0 (48.0)	48.0 (48.0)		0.119
Antibiotics prescribed	72 (84.7%)	83 (72.8%)		0.057
Urine dipstick LEU-positive	65 (76.5%)	89 (78.1%)	Bacteriuria-positive: 43 (37.7%) Bacteriuria-negative: 45 (39.5%) Error: 1 (0.9%)	0.864
Urine dipstick NIT-positive	1 (1.2%)	5 (4.4%)	Bacteriuria-positive: 5 (4.4%) Bacteriuria-negative: 0 (0.0%) Error: 0 (0.0%)	0.242
Urine dipstick LEU + NIT-positive	19 (22.4%)	20 (17.5%)	Bacteriuria-positive: 17 (14.9%) Bacteriuria-negative: 3 (2.6%) Error: 0 (0.0%)	0.471

**Table 2:** PA-100 antibiotic susceptibility test results of bacteriuria-positive samples (n = 65) for amoxicillin/clavulanic acid (AMC), ciprofloxacin (CIP), nitrofurantoin (NITRO), trimethoprim (TRI) and fosfomycin (FOS). Relative frequency is given in parentheses. S: susceptible; I: susceptible, increased exposure; R: resistant; LG: low growth (PA-100 specific warning, when AST results cannot be generated due to low growth rate of the bacterial strain); NA: not applicable refers to combinations of antibiotics and bacterial strains which EUCAST advises against. Error: technical error.

PA-100 AST	AMC	CIP	FOS	NITRO	TRI
S	43 (66.2%)	35 (53.8%)	31 (47.7%)	53 (81.5%)	49 (75.4%)
I	2 (3.1%)	7 (10.8%)	0 (0.0%)	0 (0.0%)	0 (0.0%)
R	6 (9.2%)	13 (20.0%)	14 (21.5%)	1 (1.5%)	6 (9.2%)
LG	2 (3.1%)	2 (3.1%)	5 (7.7%)	4 (6.2%)	1 (1.5%)
NA	4 (6.2%)	1 (1.5%)	6 (9.2%)	0 (0.0%)	4 (6.2%)
Error	8 (12.3%)	7 (10.8%)	5 (7.7%)	7 (10.8%)	5 (7.7%)
Total	65				

**Table 3:** Prescription of amoxicillin/clavulanic acid (AMC), cefixime and cefuroxime are combined as cephalosporine (CEPH), fosfomycin (FOS), nitrofurantoin (NITRO), ciprofloxacin (CIP), trimethoprim/cotrimoxazole (TRI/COT) and metronidazole (MET) in the control and intervention arm. Relative frequency is given in parentheses. *p*-values and odds ratios (ORs) were calculated to compare antibiotic-prescribing frequencies between the control and intervention arms.

	No Abx	AMC	CEPH	FOS	NITRO	CIP	TRI/COT	MET
Control arm								
LEU-positive	12 (14.1%)	2 (2.4%)	6 (7.1%)	41 (48.2%)	3 (3.5%)	0 (0.0%)	0 (0.0%)	1 (1.2%)
NIT-positive	0 (0.0%)	0 (0.0%)	0 (0.0%)	1 (1.2%)	0 (0.0%)	0 (0.0%)	0 (0.0%)	0 (0.0%)
LEU + NIT-positive	1 (1.2%)	1 (1.2%)	2 (2.4%)	14 (16.5%)	1 (1.2%)	0 (0.0%)	0 (0.0%)	0 (0.0%)
Total (n = 85)	13 (15.3%)	3 (3.5%)	8 (9.4%)	56 (65.9%)	4 (4.7%)	0 (0.0%)	0 (0.0%)	1 (1.2%)
Intervention arm								
LEU-positive	31 (27.2%)	8 (7.0%)	4 (3.4%)	27 (23.7%)	15 (13.2%)	2 (1.8%)	2 (1.8%)	0 (0.0%)
NIT-positive	0 (0.0%)	1 0.9%)	0 (0.0%)	3 (2.6%)	1 (0.9%)	0 (0.0%)	0 (0.0%)	0 (0.0%)
LEU + NIT-positive	0 (0.0%)	0 (0.0%)	2 (1.8%)	13 (11.4%)	4 (3.4%)	1 (0.9%)	0 (0.0%)	0 (0.0%)
Total (n = 114)	31 (27.2%)	9 (7.9%)	6 (5.3%)	43 (37.7%)	20 (17.5%)	3 (2.6%)	2 (1.8%)	0 (0.0%)
*p*-value	0.057	0.241	0.276	<0.001	0.007	0.262	0.508	0.427
OR (95% CI)	2.061 (1.0–4.6)	2.334 (0.6–13.8)	0.536 (0.1–1.8)	0.316 (0.2–0.6)	4.281 (1.4–17.9)	∞ (0.3–∞)	∞ (0.1–∞)	0 (0–29.1)

**Table 4:** Fosfomycin and other antibiotic (Abx) prescription in the control and intervention arms. Relative frequency is shown in parentheses. Bacteriuria results are from the PA-100.

	Fosfomycin	Other Abx
Control arm (n = 85)	56 (65.9%)	16 (18.8%)
Intervention arm (n = 114)	43 (37.7%)	40 (35.1%)
Bacteriuria-negative	16 (14.0%)	1 (0.9%)
Bacteriuria-positive	26 (22.8%)	39 (34.2%)
Fosfomycin–S	23 (20.2%)	8 (7.0%)
Fosfomycin–I	0 (0.0%)	0 (0.0%)
Fosfomycin–R	0 (0.0%)	14 (12.3%)
Fosfomycin–LG	0 (0.0%)	5 (4.4%)
Fosfomycin–N/A	1 (0.9%)	8 (7.0%)
Fosfomycin–Error	2 (1.8%)	4 (3.5%)
Bacteriuria error	1 (0.9%)	0 (0.0%)

## Data Availability

All data generated or analysed during this study are included in this published article.

## Abbreviations

The following abbreviations are used in this manuscript:

Abx	Antibiotic
AMR	Antimicrobial resistance
AMC	Amoxicillin/clavulanic acid
AST	Antimicrobial susceptibility test
CFU	Colony-forming unit
CI	Confidence interval
CIP	Ciprofloxacin
EAU	European Association of Urology
FOS	Fosfomycin
I	Susceptible, increased exposure
IVD	In vitro diagnostics
LEU	Leukocyte esterase
LG	Low growth
MET	Metronidazole
NA	Not applicable
NIT	Nitrites
NITRO	Nitrofurantoin
NS	Not significant
OR	Odds ratio
PC	Primary care
POC	Point of care
R	Resistant
S	Susceptible
TRI	Trimethoprim
TRI/COT	Trimethoprim/cotrimoxazole
UTI	Urinary tract infection
uUTI	Uncomplicated urinary tract infection
